# WHF Roadmap for Integrated Care in People Living with – or at Risk of – Cardiovascular Disease and Multiple Long-Term Conditions

**DOI:** 10.5334/gh.1541

**Published:** 2026-03-27

**Authors:** Laurence Sperling, Vilma Irazola, Jackie Partarrieu, Lana Raspail, Maciej Banach, Amitava Banerjee, Gene Bukhman, Maria George, Eri Toda Kato, Francisco Lopez-Jimenez, Steven Macari, Jaime Miranda, Ana Mocumbi, Pablo Perel, Dorairaj Prabhakaran, Adriana Puente Barragan, Diana Sherifali, Raul Santos

**Affiliations:** 1 Emory School of Medicine, Emory Clinic, Atlanta, USA; 2 Institute for Clinical Effectiveness and Health Policy, Buenos Aires (IECS), Argentina; 3 Independent Consultant, France; 4 WHF, Switzerland; 5 Medical University in Lodz (MUL), Lodz, Poland; 6 University College London, UCL Hospitals NHS Trust and Barts Health NHS Trust, UK; 7 Harvard University, USA; 8 INOCA International, UK; 9 Kyoto University Hospital, Japan; 10 Mayo Clinic, USA; 11 Avec France, France; 12 University of Sydney, Australia; 13 Mozambique Institute for Health Education and Research, Mozambique; 14 Center for Chronic Diseases India, India; 15 National Medical Center “20 de Noviembre” ISSSTE, Mexico; 16 McMaster University, Canada; 17 Heart Institute (InCor)/University of São Paulo (USP) Medical School, Brazil

**Keywords:** Cardiovascular disease, multimorbidity, integrated care, person-centred care, health systems

## Abstract

Cardiovascular disease (CVD) commonly coexists with multiple long-term conditions (MLTC), including diabetes, chronic kidney disease, obesity, and mental health disorders. This clustering creates a syndemic burden associated with poorer outcomes, polypharmacy, high treatment burden, and rising healthcare costs. Fragmented, single-disease care models are ill-suited to address this complexity.

The WHF roadmap for integrated care in people living with – or at risk of – CVD and MLTC provides a structured framework to support the design, implementation, and scale-up of person-centred, coordinated care models globally. Drawing on current evidence, expert consensus, case studies, and stakeholder surveys, the Roadmap outlines the epidemiological and systemic challenges of MLTC and identifies practical strategies adaptable across high-, middle-, and low-income settings.

This Roadmap emphasises multidisciplinary teamwork, aligned financing, digital health infrastructure, workforce development, patient partnership, and robust monitoring and evaluation. By shifting from siloed care to integrated, capacity-sensitive approaches, health systems can improve clinical outcomes, enhance quality of life, reduce avoidable hospitalisations, and build resilience in the face of growing multimorbidity.

## Introduction

The World Heart Federation (WHF) is committed to addressing the global burden of cardiovascular disease (CVD) through evidence-based strategies, advocacy, and collaboration.

In 2014, WHF launched its Roadmaps initiative (1) to support national efforts aligned with the United Nations Sustainable Development Goal of reducing premature mortality from non-communicable diseases (NCDs), including CVD, by 30% by 2030.

As part of this initiative, WHF has developed the *Roadmap for Integrated Care in People living with or at risk of Cardiovascular Disease and Multiple Long-Term Conditions (MLTC)*. This Roadmap uses the term *multiple long-term conditions* (MLTC) rather than alternatives such as multimorbidity or clusters of systemic disorders to emphasise a person-centred perspective. MLTC refers to the coexistence of two or more ongoing health conditions (physical, mental, or infectious) lasting at least six months with no condition designated as primary (2,3). This framing recognises that conditions interact over time and shape individuals’ daily lives, priorities and care needs. It moves beyond viewing diseases as isolated disorders and instead underscores the importance of coordinated, continuous and holistic care that addresses the whole person. MLTCs are increasingly prevalent worldwide and constitute a major and growing public health concern (4).

Recent WHF surveys of healthcare professionals and people living with cardiovascular disease underscore the need for integrated care approaches (Section IV). Both groups highlighted the high prevalence of comorbid conditions such as diabetes, obesity, kidney disease, and mental health disorders, alongside systemic barriers including fragmented care and lack of coordination. These findings provide strong justification for a roadmap to support integrated models of care globally.

People living with MLTC frequently experience a high burden of symptoms and treatment demands, fragmented and poorly coordinated care, polypharmacy, poorer health-related quality of life and clinical outcomes, and significantly increased healthcare costs (5).

CVD frequently coexists with other chronic conditions such as diabetes, hypertension, dyslipidaemia, metabolic syndrome, obesity, depression, and chronic kidney disease. These overlapping conditions require a shift from disease-specific approaches to integrated care: a more holistic, coordinated, and person-centred model better suited to the needs of people with complex health profiles.

This roadmap builds on WHF’s growing series of publications addressing key issues in cardiovascular health, including the *Roadmap on the prevention of cardiovascular disease among people living with diabetes*, and those on atrial fibrillation, secondary prevention of CVD, heart failure, cholesterol, digital health in cardiology, rheumatic heart disease, tobacco control and single pill combination therapies. These resources have helped guide policymakers and health systems in designing strategic responses to specific CVD-related challenges.

The development of this Roadmap also reflects WHF’s commitment to co-creation and meaningful engagement with people living with CVD and MLTC. The writing group included patient representatives and drew on direct patient input, recognising the essential role of patient organisations in shaping policies, improving care delivery, and supporting the translation of evidence into practice. Patient groups are key partners in advocating for person-centred, integrated care and in helping ensure that health system reforms reflect the real-world needs and priorities of those most affected.

With health systems increasingly encountering people with multiple chronic conditions, there is a pressing need for practical steps to implement integrated care. This roadmap offers a framework for understanding and addressing the challenges faced by people living with CVD and MLTC. It promotes sustainable and adaptable solutions to improve health outcomes and quality of life for these patients, as well as equity and better quality of care.

This Roadmap is designed not only to identify immediate priorities for improving integrated care in people living with CVD and MLTC, but also to support long-term sustainability and scalability. By highlighting system-level facilitators, real-world considerations, adaptable implementation strategies, and lessons learned across diverse settings, it provides a framework that countries and stakeholders can use to develop, evaluate, and progressively scale integrated care models in ways that meet the needs of individuals, are feasible, context-specific, and resilient over time.

## Purpose of the WHF Roadmap

This *WHF Roadmap for integrated care in people living with – or at risk of – CVD and MLTC* provides a structured approach to advancing integrated care globally. It draws on current evidence and practice, identifies gaps and systemic barriers, and proposes actionable, context-specific strategies for designing, implementing, and scaling integrated models of care.

The roadmap is intended to support a wide range of stakeholders (including healthcare professionals, health system leaders, policymakers, people living with CVD and MLTC, caregivers and the community) in building more equitable, sustainable, and person-centred care systems.

Ultimately, this roadmap illustrates how integrated care can transform both the experience and outcomes of people living with CVD and MLTC, while also strengthening health systems to respond more effectively to the growing burden of multiple coexisting chronic conditions.

## Top 10 take-home messages

### What every stakeholder should know about integrated care for CVD and MLTC

1. **Integrated care is essential**
  Cardiovascular disease rarely occurs alone. Most patients also live with multiple long-term conditions. Integrated care models are necessary to manage this complexity effectively, improve outcomes, and reduce the strain on health systems.
2. **People-centred approaches are vital**
  Integrated care puts individuals – not diseases – at the centre. Care must align with people’s needs, priorities, and capacity to manage their treatment burden. It must also include caregivers and acknowledge the support networks that contribute to health and wellbeing.
3. **One size does not fit all**
  Integrated models must be tailored to each context and responsive to the diverse cultural, structural, and resource-related realities that exist across – and within – countries and regions. Strategies differ according to local needs and capacities, but share a common goal: accessible, coordinated, continuous, and person-centred care.
4. **The SELFIE framework provides structure**
  The SELFIE framework (*Sustainable intEgrated chronic care modeLs for multi- morbidity: delivery, FInancing, and performance*) outlines six components – service delivery, workforce, leadership, financing, technology, and monitoring – that support integrated care implementation. However, true people-centredness requires a seventh component: partnership with patients, caregivers, and patient associations. Their co-leadership in designing and evaluating care ensures services are relevant and responsive.
5. **Multidisciplinary teams are the foundation**
  Effective collaboration among specialist physicians, primary care clinicians, nurses, pharmacists, community health workers (CHWs), social services, behavioural health specialists, and caregivers is essential for delivering coordinated, person-centred care. Training and support for teams must foster shared responsibility and mutual respect.
6. **Digital health and data-sharing support continuity of care and personalisation**
  Technology – including interoperable electronic health records (EHRs) and mobile health (mHealth) tools – enhances care coordination, enables tailored interventions, supports patient self-management, and facilitates real-time monitoring and learning.
7. **Roadblocks must be addressed head-on**
  Legal, governmental, organisational, cultural, and financial constraints limit progress. Resistance to change among healthcare professionals and systems remains one of the most significant obstacles. Overcoming these barriers requires leadership, investment, supportive regulation (e.g. scope-of-practice laws), education, and alignment of incentives that reward collaborative, integrated care.
8. **Monitoring and evaluation (M&E) must focus on what matters most**
  Evaluation should go beyond service metrics to include what matters most to people, such as quality of life, care experience, and capacity to manage one’s health. Tracking implementation, service performance, and patient-reported outcomes (PROs) ensures accountability and enables adaptation. Embedding these processes within a learning health system approach allows continuous feedback and improvement. Clear key performance indicators (KPIs) help systems learn and evolve.
9. **Community involvement enhances sustainability**
  High-quality health systems are people centred. Engaging patients, caregivers, and communities in the co-design, delivery, and governance of care builds trust, ensures relevance, and strengthens long-term sustainability. This inclusive approach aligns with global frameworks for effective, resilient care systems.
10. **The time to act is now**
  The COVID-19 pandemic exposed critical global and health system vulnerabilities, emphasising the need for integrated, adaptable care. Integrated care is not only a reform strategy, but also the foundation for building resilient, equitable, and future-ready healthcare systems able to manage rising MLTC.

## I. Understanding the burden

### 1. Introduction to cardiovascular disease and multiple long-term conditions

Despite major advances in prevention, diagnosis, and treatment over recent decades, CVD and accompanying cardiometabolic conditions remain a leading cause of morbidity and mortality globally (6). Its aetiology is complex, shaped by metabolic, behavioural, genetic, social, and environmental factors. CVD is frequently interconnected with other health conditions in a bidirectional manner, with each condition worsening the other, creating a vicious cycle that intensifies disease burden (7).

At the same time, medical advances, changes in global public health dynamics, and ageing populations have contributed to a sharp rise in the number of individuals living with MLTC (8). This trend is just as evident in high-income countries (HICs) as in low- and middle-income countries (LMICs), although the nature and drivers of multimorbidity may vary locally and globally. MLTC are associated with reduced quality of life, increased healthcare costs, polypharmacy, lower treatment adherence, and clinical complexity, posing challenges for health systems regardless of income setting (5,9,10).

Obesity is a major contributor to MLTC worldwide (7,11). It serves as a common driver for dysglycaemia, type 2 diabetes, atherogenic dyslipidaemia, low-grade inflammation, sleep apnoea, and suboptimal physical activity, in addition to chronic kidney and liver diseases. Obesity also worsens cardiovascular outcomes, especially in heart failure with preserved ejection fraction, atrial fibrillation, and hypertension (12).

Infectious diseases, whether acute (e.g. COVID-19, influenza) or chronic (e.g. HIV, tuberculosis, Chagas disease), can further complicate care, particularly in settings where infectious and non-communicable disease burdens coexist (13,14,15).

Mental health disorders, most notably depression, are common across income settings and have strong bidirectional links with CVD. Depression not only increases the risk of cardiovascular events but also undermines treatment adherence and lifestyle changes (16,17). These challenges are compounded by social deprivation, health inequities, and systemic barriers to accessing care (18).

### 2. The syndemic of CVD and MLTC

Cardiovascular disease is central to the burden of multimorbidity. The Cardio-Kidney-Metabolic (CKM) syndrome, recently defined by the American Heart Association (7), encapsulates the interconnected nature of CVD, chronic kidney disease (CKD), and metabolic disorders such as obesity and type 2 diabetes (19). These conditions frequently co-occur, and their interaction accelerates disease progression and worsens outcomes.

A recent global modelling study further strengthens the syndemic framing of CVD and its interconnected risk factors. Chong et al. (20) project the global burden of CVD from 2025 to 2050, attributing it to five key modifiable risk factors: high systolic blood pressure, high LDL cholesterol, elevated fasting plasma glucose, high body mass index (BMI), and tobacco use. These risk factors – frequently co-occurring and socially determined – are not isolated epidemics but a global syndemic, collectively driving the incidence of CVD and premature mortality.

In 2050, high blood pressure alone is expected to account for 17 million deaths, with millions more from high LDL cholesterol, high fasting plasma glucose, obesity, and tobacco. Despite projected reductions in age-standardised disability-adjusted life year (DALY) rates for some risk factors, the absolute burden will remain immense due to population growth and ageing. Importantly, the authors emphasise the need for integrated, upstream prevention strategies rather than siloed risk-factor management. This supports a life-course approach, recognising that CVD and metabolic risk begins in utero and is shaped by early-life exposures, obesogenic environments and social determinants from childhood through adolescence, early adulthood and across the lifespan.

Recent research also highlights the importance of understanding how MLTC evolve over time. A large observational study by Han et al. (21) used machine learning and causal inference methods to map MLTC progression among 190 diseases in over 500,000 participants from the UK Biobank. Their findings reveal that multimorbidity is not random but tends to follow predictable patterns across disease clusters, with key chronic conditions such as CVD acting as both influencers and recipients of disease progression. Importantly, the study identifies sex-specific multimorbidity “constellations”, showing how clustering tendencies differ between males and females.

In males, progression pathways were more strongly driven by cardiometabolic conditions, with CVD occupying a particularly central role within disease networks. In females, multimorbidity patterns showed greater clustering around immune-mediated, musculoskeletal, endocrine, and mental health conditions, with more complex and interconnected trajectories over time.

**Figure 1:**
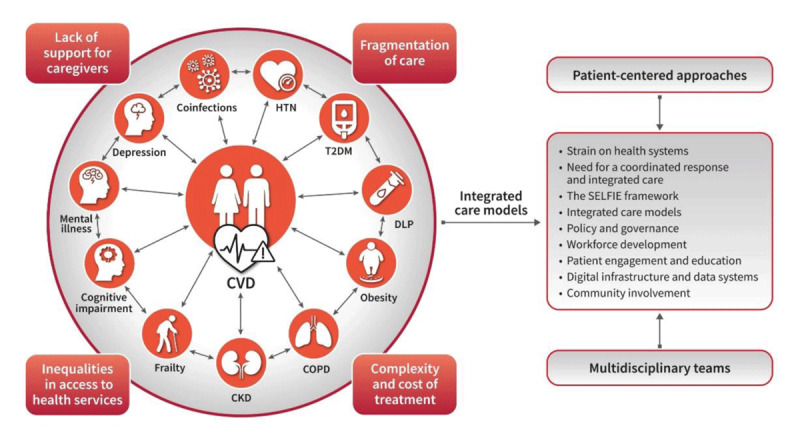
The Global Syndemic of Cardiovascular Disease and Multiple Long-Term Conditions. Patients with CVD lives with MLTC (left). Integrated care models are necessary to manage this complexity effectively, improve outcomes, and reduce the strain on health systems (right). CVD: cardiovascular disease; MLTC:multiple long-term conditions; HTN: hypertension; T2DM: type 2 diabetes mellitus; DLP: dyslipidemia; COPD: chronic obstructive pulmonary disease; CKD: chronic kidney disease; SELFIE: Sustainable intEgrated care modeLs for multi-morbidity: delivery, FInancing and performancE.

These insights highlight the potential of technology-enabled, data-driven approaches to identify sex-specific disease patterns and inform cross-specialty, targeted actions for integrated care. While these associations require validation across diverse populations, mapping out these clusters is crucial to addressing the rising challenge of MLTC and developing effective, tailored interventions. Figure 1 illustrates the syndemic of living with CVD and MLTC and the need for integrated care models.

Managing people with CKM syndrome requires integrated, person-centred care. Clinicians treating CVD must also address conditions such as obesity, hypertension, dyslipidaemia, sleep apnoea, dysglycaemia, metabolic dysfunction-associated steatotic liver disease (MASLD), and CKD, while non-cardiovascular specialists need to evaluate and manage cardiovascular risk. Early identification of risk and coordinated, disease-modifying interventions can reduce morbidity, improve quality of life, and extend survival.

### 3. Burden of care

#### i. A widespread and growing challenge

The global burden of NCDs continues to rise in all regions of the world. Hypertension (22), diabetes (23), and cardiovascular disease are among the most prevalent and life-threatening conditions. In 2022, 828 million adults were living with diabetes mellitus (DM) (22,24), a dramatic increase since 1990. Each year, over 15 million people between ages 30 and 69 die prematurely from NCDs, with 85% of these deaths occurring in LMICs (25,26).

In high-income settings, the rising prevalence of MLTC is driven by ageing populations, sedentary lifestyles, unhealthy diets, and increasing mental health concerns. Up to one in four adults in HICs live with multiple chronic conditions, often requiring complex, multidisciplinary management (27). The modelling by Chong et al. illustrates how this burden will grow in the coming decades. By 2050, high blood pressure, obesity, diabetes, and other interrelated risk factors will continue to dominate global CVD mortality. These trends highlight the urgency of moving from fragmented chronic disease management to integrated care systems capable of simultaneously addressing multiple upstream determinants (28).

Mental health disorders such as depression are particularly prevalent among people with MLTC (29) and are associated with worse outcomes across settings. The *Study on Global Ageing and Adult Health*, conducted in six LMICs including Ghana and South Africa (27), showed a dose-dependent increase in depression with the number of chronic diseases, a finding mirrored in HIC population studies.

#### ii. The human cost

People living with CVD and MLTC face compounded physical, emotional, and social challenges. For example, CKD, poorly controlled diabetes, and other conditions can accelerate the progression of heart failure, while mental health disorders such as depression (up to three times more common in this population) are associated with poorer adherence to treatment, increased risk of hospitalisation and complications, income loss, and diminished quality of life.

Beyond the clinical implications, MLTC often bring a significant human toll. Individuals may experience physical limitations that affect daily functioning, emotional distress from living with multiple conditions, social isolation, and economic insecurity due to time off work or costly healthcare expenses. These pressures extend to caregivers, who may shoulder the responsibility of navigating complex systems and supporting daily disease management (30).

Polypharmacy frequently becomes necessary, particularly when managing CVD alongside MLTC. Although often beneficial, the need for multiple medications may increase the risk of drug interactions and overall treatment burden. Overlapping prescriptions – especially among cardiovascular agents, antidiabetic medications, and antidepressants – require careful coordination. Unfortunately, such coordination is often lacking in fragmented health systems, where people encounter multiple healthcare professionals who may not communicate effectively with one another (31).

The situation is especially difficult for people in rural or underserved areas, where access to specialised services is limited and pathways of care are often unclear. These patients must manage complex treatment regimens with minimal support, leading to poorer outcomes and potentially avoidable complications.

**Case study: Integrated care for cardiometabolic-renal disease and depression in India**

Context

A man in his late 60s was seen at a cardiology clinic in a semi-urban community in India. Care in this setting is typically driven by specialty and subspecialty services, especially in the private sector, while the public sector faces major resource pressures. This person had experienced both systems.

There was limited coordination between cardiology, diabetes, renal, and mental health services, and almost no effective primary care. As a result, patients often moved between multiple providers without continuity, clear communication, or shared plans. This led to delays, repeated tests, and unnecessary costs. Better integration was needed to allow individuals with complex cardiometabolic–renal conditions and depression to receive care closer to home, with fewer appointments and clearer pathways.

Problem

The challenge was to create an integrated community-based service for cardiometabolic-renal disease and depression, supported by pharmacy input. The goal was to reduce the number of specialist appointments and number of hospital admissions for people with these combined conditions.

Approach

The first step was to map available staff across primary care, cardiology, diabetes, renal medicine, psychiatry, nursing, physiotherapy, and pharmacy. A monthly multidisciplinary team meeting was considered but rejected due to limited capacity. A second option – a fully community-delivered service – was also set aside because it would have required substantial training.

The final strategy was a weekly, community-based primary care clinic where relevant specialists visited on a fixed day so that patients could have all appointments on the same day. Community health workers (known as ASHAs in India) were trained to ask about symptoms and perform simple checks for heart disease, diabetes, and depression. Individuals identified by CHWs were directed to the primary care centre, where specialists were available. Referral to secondary or tertiary hospitals occurred only when necessary. The number of specialist appointments and admissions were tracked for three months before and three months after implementation.

Results

The new model was introduced over two months and then continued routinely. Hospital admissions fell by 18% compared with the pre-intervention period. Specialist clinic appointments (cardiology, diabetes, renal, psychiatry) decreased by 13%. Satisfaction was high: 87% of staff and 91% of patients reported positive experiences with the new model of care.

Lessons and recommendations

A community-based integrated clinic for cardiovascular disease, diabetes, and depression can be established using existing resources. Outpatient visits and hospital admissions decreased, and the model was acceptable to both patients and clinicians.

Key recommendations:

- Use community health workers to support early identification and triage.
- Bring specialists into the community on a predictable schedule.
- Use simple monitoring (e.g. pre/post counts of visits and admissions) to measure impact.
- Prioritise continuity and communication across specialties.

Integrated care for cardiometabolic-renal disease and depression is feasible and effective for improving care, as in the case above, even in resource-pressured settings.

*Surveillance of noncommunicable diseases by community health workers in Kerala: the epidemiology of noncommunicable diseases in rural areas (ENDIRA) study. Menon J, Joseph J, Thachil A, Attacheril TV, Banerjee A. Glob Heart. 2014 Dec;9(4):409–17. doi: 10.1016/j.gheart.2014.07.003. Epub 2014 Nov 22. PMID: 25592794*

**The rising number of individuals living with MLTC presents significant challenges for health systems globally, including:**

- **Increased care complexity:** Involvement of multiple specialists can lead to duplication, conflicting advice, and confusion for patients.
- **Higher healthcare costs:** Frequent consultations, diagnostic tests, polypharmacy, and unplanned hospital admissions raise direct and indirect costs.
- **Systemic inequities:** In many LMICs, health systems remain focused on acute care and infectious diseases (although they often coexist with MLTC) leaving major gaps in chronic disease management (32). The coexistence of NCDs with endemic infections such as HIV, tuberculosis, and Chagas disease (15) further complicates clinical care and resource allocation.

This convergence of chronic and infectious diseases, particularly in LMICs, exacerbates the strain on already overstretched healthcare services, increases the risk of adverse health outcomes, and heightens health inequalities (33).

#### iii. Strain on health systems

Both HIC and LMIC health systems are struggling to adapt to the realities of MLTC. In LMICs, challenges are often related to limited resources, fragmented services, and competing health priorities such as infectious diseases and maternal/child health. In these settings, multimorbidity frequently reflects a combination of cardiometabolic disease, persistent infections, and undernutrition, creating a unique and complex burden (34,35,36). Population ageing is a key driver of the growing global burden of CVD and MLTC. As life expectancy rises, more people live long enough to develop chronic diseases and to experience MLTC, increasing the need for ongoing, coordinated care and placing greater demands on health systems worldwide. Meanwhile, in HICs, health systems face pressure from increasing costs, workforce shortages, and the need to redesign services to move beyond single-disease specialisation (37).

Fragmented care pathways, siloed health records, and lack of coordination among healthcare professionals are common challenges in both contexts, leading to duplication, delays, polypharmacy risks, and suboptimal outcomes (38). People living with MLTC (particularly those with severe symptoms or living in rural areas) may struggle to navigate multiple appointments, therapeutic regimens, and healthcare settings, resulting in reduced adherence and avoidable hospitalisations (39).

The paradox of available treatments but poor implementation highlights the systemic failures in CVD and MLTC care (40). High rates of preventable mortality are due to inadequate patient support, leading to missed early interventions for hypertension, diabetes, and dyslipidaemia. Lack of coordinated care results in medication conflicts and duplicate testing, further complicating treatment adherence.

The omission to address psychosocial burdens that impact adherence contributes to care fragmentation and unmet healthcare needs. “Treatment burden is not just a patient problem; it is a system failure that leads to preventable deaths,” say Boehmer et al (41).

#### iv. The need for a coordinated response

The increasing prevalence and complexity of MLTC in those living with CVD demand a shift towards integrated care models and value-based health systems (42) that prioritise continuity, personalisation, and efficient use of resources. These models must be adaptable to all resource settings: from highly specialised systems in HICs to primary care-based networks in LMICs (43).

The ideal scenario involves multidisciplinary care teams, bringing together medical and allied health professionals, working in concert to provide streamlined, person-centred care. While full integration remains aspirational in many countries, there are concrete opportunities for progress through better coordination between services, clearer referral pathways, and shared decision-making with patients (44).

To support this, health professionals across disciplines need to be trained to recognise coexisting conditions using accessible diagnostic tools and to apply evidence-based therapies that address multiple conditions simultaneously. Integrated clinical guidelines, interoperable digital health systems, and policies that encourage task-sharing can help build the foundations of more coherent care.

Concerted action across sectors, combining health, social care, and community support, is essential to respond to the growing human and systemic burden of CVD and MLTC. Without such action, the gap between patient needs and service delivery will continue to widen, with serious consequences for individuals, families, and societies.

#### v. A call for integrated care

The way forward for health systems facing increasing pressure from CVD and MLTC is to pivot towards integrated, person-centred care models that address physical, mental, and social health needs holistically (45). This includes interdisciplinary collaboration across cardiology, nephrology, endocrinology, psychiatry, and primary care, as well as with allied health professionals and community-based services. As noted by Chong et al., “The burden is more than the sum of its parts.”

Syndemic thinking and strategy allow health systems to move beyond isolated disease management and embrace integrated solutions that tackle the shared societal drivers of chronic disease risk. Prioritising upstream, preventive interventions, especially among younger populations, will be crucial for reversing the long-term trends in CVD-related mortality and morbidity.

In LMICs, there is a need for stronger primary healthcare, better care coordination, and improved access to diagnostics (46,47,48,49). The COVID-19 pandemic exposed the fragility of health systems worldwide, but especially in LMICs, where people with chronic diseases experienced major disruptions in care (50). The crisis also underscored the value of digital health tools, interoperable health information systems, and real-time data-sharing to support continuity of care – levers which are equally relevant in HICs, where digital transformation and system integration remain incomplete (51).

Integrated care models are especially critical for addressing polypharmacy (52), improving treatment adherence (53), and ensuring that care is not only clinically effective but also accessible, equitable, and respectful of patient preferences and socioeconomic contexts (54).

These challenges also underscore the importance of strengthening health systems in line with the principles of universal health coverage (UHC), defined by the World Health Organization as ensuring that all people have access to the health services they need, when and where they need them, without financial hardship (55). Achieving UHC requires health systems that are not only accessible but also coordinated, equitable, and capable of addressing complex, long-term care needs.

#### vi. The broader impact

MLTC have far-reaching effects beyond health outcomes. They impact emotional wellbeing, economic stability, work productivity, and family life. In both HICs and LMICs, caregivers shoulder a significant burden, often without adequate support. Younger, working-age adults with MLTC face unique challenges, including lost income and delayed care-seeking due to work or family obligations.

Improving care for people with MLTC is therefore a societal as well as a healthcare imperative (56). Ensuring access to integrated services – across prevention, diagnosis, treatment, and rehabilitation – can reduce inequalities, boost workforce resilience, and make healthcare more sustainable. As the impact of MLTC continues to grow, health systems worldwide must adapt to provide better, more connected care to those who need it most.

## II. The case for integrated care

### 1. Definition and scope

Integrated care is increasingly recognised as a critically needed response to the rising prevalence and complexity of MLTC, particularly in people with CVD. At its core, integrated care refers to the coordinated delivery of health services that are continuous, person-centred, and responsive to the full spectrum of patient needs, across disciplines, healthcare professionals, and settings (57).

Rather than focusing on individual diseases in isolation, integrated care seeks to provide a seamless experience for patients by linking primary care, specialists (e.g. cardiologists, nephrologists, endocrinologists), mental health professionals, pharmacists, and social services within a single, coordinated framework. This approach supports more holistic, efficient, and equitable healthcare delivery (58).

Growing recognition of the overlap between cardiovascular disease and other LTCs is reflected in a recent consensus document on cardiovascular-liver-metabolic health. Chew and colleagues (59) convened a multidisciplinary panel of cardiologists, hepatologists, and endocrinologists to develop consensus recommendations on the identification and management of MASLD in people with or at risk of CVD. Their Delphi-based guidance underscores the need for earlier identification of MASLD in cardiovascular populations, and closer alignment between cardiovascular and metabolic care pathways. This work illustrates a broader shift towards evidence-informed, cross-specialty approaches to managing the multisystem consequences of cardiometabolic disease.

**Integrated care encompasses:**

- Multidisciplinary collaboration, ensuring all relevant professionals contribute to shared care plans.
- Continuity and personalisation of care, with ongoing follow-up and adjustments.
- Shared decision-making, recognising patient preferences and life context.
- Care coordination mechanisms, such as nurse-led teams or digital platforms.
- Proactive use of PROs to inform treatment and monitor burden (60,61).

Despite widespread endorsement of its value, implementation remains inconsistent. As detailed in Table 1, barriers range from poor interoperability of digital systems and fragmented funding, to gaps in training, regulation, and cultural alignment across care teams (62).

**Table 1:** Roadblocks to integrated care.


DOMAIN	ROADBLOCK	DESCRIPTION	MITIGATION STRATEGIES

Administration/regulation	Regulatory & legal constraints	Data-sharing restrictions, authorisation process, liability issues, administrative burden.	Review and harmonise regulations across sectors. Develop shared data-governance frameworks. Enable cross-organisational contracting and pooled budgets.

	Legacy institutional structures, Lack of political priorisation	Long-standing fragmentation between health and social care, primary and secondary care, etc.	Create joint governance bodies across sectors. Establish regional/territorial “integration boards” with shared accountability. Promote cross-sector strategic planning.

Funding	Fragmented or inadequate funding	Siloed budgets: incentives that reward activity rather than outcomes or collaboration.	Introduce funding models that incentivise coordination (bundled payments, capitation, population-based funding). Pilot shared financing schemes across organisations. Ensure stable funding for coordination roles.

	Lack of funding for coordination/case management	Non-clinical but essential tasks (navigation, social support) undervalued or unfunded.	Reimburse care coordination and case management. Invest in integrated care teams (nurse coordinators, social workers). Include social care explicitly in health budgeting frameworks.

Inter-organisational domain	Poor digital interoperability	Multiple incompatible IT systems; no shared EHR; difficult information exchange.	Invest in shared digital platforms and interoperable standards. Mandate vendor-neutral interoperability. Provide national frameworks for EHR sharing and data quality standards. Invest in digital literacy for HCPs, CHWs, patients and caregivers.

	Lack of shared goals, vision, trust	Organisations prioritise internal goals; competition; absence of shared leadership.	Develop shared vision/mission statements. Establish joint KPIs and integrated outcomes reporting. Create inter-organisational leadership forums.

Organisational domain	Cultural, professional & hierarchical differences	Differing training, values, norms, power	Deliver inter-professional education and collaborative leadership training.


Based on the findings of *Barriers to the Integration of Care in Inter-Organisational Settings: A Literature Review (Auschra 2018)* and complementary literature.

### 2. Patient capacity and treatment burden

The growing population of people with CVD and MLTC has increasingly complex care needs. Managing multiple conditions often involves interacting with several healthcare professionals, adhering to intensive medication regimens, implementing lifestyle changes, and navigating bureaucratic or logistical obstacles. This workload is referred to as the treatment burden (60,63).

**Figure 2:**
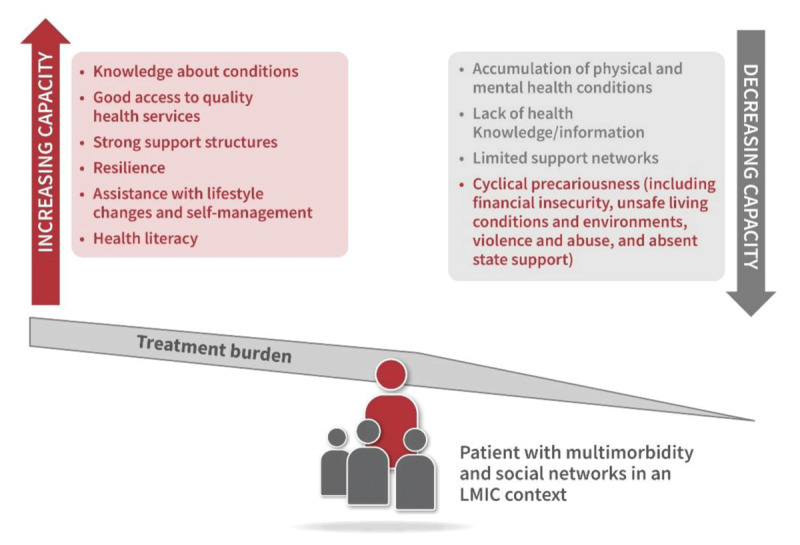
Treatment burden and reduced patient capacity. Patient capacity is shaped by access to quality health services, knowledge, social support, resilience, and assistance with self-management. In contrast, accumulated physical and mental health conditions, limited information, weak support networks, and cyclical socioeconomic precarity reduce capacity. When treatment burden exceeds capacity, patients experience increased risk of poor adherence, complications, and adverse outcomes.

Patient capacity, in turn, refers to personal resources available to manage this burden. These include cognitive function, health literacy, emotional resilience, physical health, financial resources, access to support networks, and digital access. When treatment burden exceeds capacity, adherence drops, complications rise, and outcomes worsen (64), as demonstrated in Figure 2.

As Mendoza-Quispe et al. (60) state: “Treatment burden is a key determinant of patient adherence and engagement in care, yet it is often overlooked in clinical practice and research.” Boehmer et al. (64) further emphasise the fluid nature of patient capacity, which may vary with illness episodes, stress, and changes in social circumstances.

### 3. Impact on outcomes

High treatment burden combined with low capacity is associated with:

- Poor medication adherence and missed follow-ups.
- Increased depression, anxiety, and disengagement from care.
- Higher risk of hospital admissions and premature death.
- Reduced quality of life and economic hardship for patients and families.

People living with CVD and MLTC face specific challenges including polypharmacy, overlapping side effects, and conflicting advice from different healthcare professionals. Common regimens may involve antihypertensives, anticoagulants, statins, glucose-lowering drugs, and other medications, each requiring careful titration, monitoring, and coordination. The WHF Roadmap on Single Pill Combination Therapies highlights that complex medication regimens and high pill burden are major barriers to adherence and effective cardiovascular prevention and management, and emphasises the importance of strategies that simplify treatment and improve coordination of care (65).

Reproduced from Pinxteren et al. (2023), Frontiers in Medicine, under CC BY 4.0 license.

As mentioned in the introduction, the WHF patient survey (see Section IV) revealed that 76% take five or more medications, yet only 9% had access to integrated risk assessment tools. Coordination of care was rated as poor or very poor by over a third (36%), while only one in five felt their care was well coordinated. This disconnect illustrates the heavy treatment burden faced by patients and the inefficiencies inherent in siloed systems.

**Case study: Managing five or more prescriptions after a cardiovascular event in Brazil**

Context

This case concerns a 62-year-old man living in São Paulo who was recently discharged after a non-ST elevation myocardial infarction (NSTEMI) treated with two pharmacological stents. He has type 2 diabetes (10 years), obesity, long-standing hypertension, and hyperlipidaemia. Like many patients in large urban settings, he must manage several chronic conditions at the same time. This case matters because it shows the everyday difficulties that patients face when taking multiple medicines, despite clear clinical guidelines. It highlights how complex treatment plans, overlapping dosing schedules, and worries about side effects can make it hard for individuals to maintain good long-term control of cardiovascular and metabolic risk factors.

Problem

After discharge, he was prescribed six medications: atorvastatin, metformin XR, ramipril, bisoprolol, aspirin, and clopidogrel. He frequently missed doses because he felt unsure about timing and experienced side effects. As a result, his LDL cholesterol remained high (2.4 mmol/L), blood pressure was not controlled (148/90 mmHg), and HbA1c stayed above target (8.2%). The challenge was to ensure adherence to treatment in order to meet clinical goals and reduce the risk of recurrent cardiovascular events.

Approach

The care team completed a full medication review and discussed options with the patient, including combination pills and digital reminders. They chose a “medication simplification” approach: switching to combined therapies when possible and providing clear, structured education about why each medicine was needed. His pharmacist was involved to reinforce key messages at every refill, and he joined a nurse-led cardiovascular risk clinic for regular follow-up. Other options – such as mobile phone reminders or pill-organiser systems – were considered but abandoned because he was not comfortable with digital tools. The final model aimed to reduce the complexity of his regimen, increase his understanding of his treatment, and offer consistent support from different members of the healthcare team.

Results

After three months, adherence improved from about 60% to 85% of prescribed doses. His LDL cholesterol fell from 2.4 to 1.4 mmol/L after switching to rosuvastatin/ezetimibe combination therapy. Blood pressure improved to 136/82 mmHg, and HbA1c decreased to 7.5%. Some missed doses still occurred, mostly at weekends. Structured education and pharmacist involvement were the most effective elements, while the simplified medication schedule helped but did not fully facilitate adherence.

Summary of key changes

**Table:** 

Metric	Pre-intervention	Post-intervention
Medication adherence	~60%	~85%
LDL cholesterol (mmol/L)	2.4	1.4
Blood pressure (mmHg)	148/90	136/82
HbA1c (%)	8.2	7.5

Lessons and recommendations

Medication simplification and clear patient education are essential for improving adherence in people living with MLTC. Pharmacist involvement provides an important additional layer of support. Regular structured follow-up (such as nurse-led clinics) helps identify problems early. Digital tools may be useful but should be adapted to the patient’s abilities and preferences. Even with good interventions, perfect adherence may not be possible. Ongoing support, flexibility, and simple monitoring are key to maintaining long-term control of cholesterol, blood pressure, and diabetes.

Treatment burden also has economic implications: increased hospital use, higher system costs, and productivity losses due to work interruptions or early retirement. Reducing this burden can improve both patient outcomes and system sustainability.

Efforts to prioritise outcomes that matter most to people are particularly important in low-resource settings, where healthcare access is uneven, and out-of-pocket expenses often limit adherence. The COSMOS study, a global initiative led by Vidyasagaran et al. (66), developed core outcome sets (COS) for multimorbidity prevention and treatment trials in low- and middle-income countries. These COS – focused on treatment adherence, adverse events, out-of-pocket expenditure, and quality of life – reflect the real-world priorities of patients, caregivers, clinicians, and policymakers across 33 countries. This work reinforces the need for context-specific, person-centred outcomes in trials and health service planning, particularly for those living with the compounded burdens of CVD and MLTC in resource-constrained environments.

The POLY-HF trial (67) showed that simplifying treatment regimens can meaningfully reduce treatment burden and improve patient capacity in socioeconomically disadvantaged people with heart failure. A once daily polypill improved adherence, quality of life and improved clinical outcomes compared with usual care, with strong patient acceptability driven by reduced pill burden and fewer missed doses.

### 4. Global perspectives

As already mentioned, treatment burden manifests differently across settings:

- In HICs, individuals may have access to advanced care but experience fragmented services due to siloed specialties and lack of coordination.
- In LMICs, access to essential medications, diagnostics, and continuity of care is often limited. People frequently travel long distances, face high out-of-pocket costs, and may lack the digital tools or health literacy to self-manage effectively (60).

Addressing both sides of the equation – minimising unnecessary workload while strengthening patient capacity – is essential to avoid the preventable morbidity and mortality associated with poorly managed MLTC.

### 5. Benefits of integrated care

#### i. Improved outcomes and quality of life

Integrated care has been shown to improve outcomes and quality of life for people living with CVD and MLTC. By replacing fragmented, condition-centred services with a coordinated, person-centred approach, it allows for healthcare delivery that is continuous, comprehensive, and aligned with individuals’ needs and capacities. Although the impact of integrated care can be difficult to measure – given the complexity and variability of such interventions, which are not easily evaluated through traditional trial designs – the evidence consistently points to better coordination, more proactive management, and improvements in both clinical indicators and people’s lived experiences.

Patients enrolled in integrated care pathways frequently report greater satisfaction due to the accessibility, responsiveness, and personalisation of care. Data shows that integrated care can reduce hospital admissions and readmissions by enabling early identification of clinical deterioration, streamlining referrals, and ensuring timely follow-up (68). By requiring collaboration within multidisciplinary teams (comprising cardiologists, general practitioners, pharmacists, dietitians, mental health professionals, and other specialists and allied professionals), integrated care also mitigates the risk of conflicting treatment plans and adverse drug interactions (69).

A key feature of successful integrated care models is the use of Patient Reported Outcome Measures (PROMs) (70) to assess the burden of treatment and adapt care accordingly. Instruments such as the *Treatment Burden Questionnaire* (TBQ) help to identify those struggling to manage complex regimens, enabling clinicians to respond with capacity-adjusted interventions. As Mendoza-Quispe et al. argue (60), integrating PROMs into cardiovascular care can enhance person-centred care and long-term outcomes. Regular screening for treatment burden allows healthcare teams to engage in meaningful shared decision-making, ensuring that care plans are both clinically effective and realistic for the individuals.

Interventions such as nurse-led case management, patient navigators, care coordination platforms, simplified medication regimens, and the use of digital health tools have been shown to further enhance the patient experience. Telemedicine and mHealth technologies play a growing role in improving care accessibility and supporting self-management, especially for people with mobility challenges or those living in remote areas. These tools offer flexible, real-time communication and monitoring, contributing to better disease control and quality of life (71).

By addressing not only medical needs but also psychosocial and emotional factors, integrated care supports people holistically. This reduces confusion, improves continuity, and strengthens trust in healthcare professionals. Patients receiving integrated care are more likely to feel empowered, participate in their own care decisions, and develop sustained relationships with their clinical teams, all of which contribute to better outcomes.

**Case study: Nurse-led integrated care for people with CVD and CKD**

Context

This case is from the Heart-Nephrology-Diabetes (HND) Centre at Danderyd University Hospital in Sweden. Since 2013, the Centre has delivered integrated, person-centred care for people living with CVD, CKD, and diabetes. All staff receive training in person-centred and multidisciplinary care, ensuring a consistent approach across cardiology, nephrology, and diabetes teams. The clinic provides an ideal setting to test new care models because patients have complex multimorbidity and are at high risk of poor outcomes. This case assesses whether a nurse-led integrated care team can improve outcomes and patient experience compared with traditional, separate specialist clinics.

Problem

CVD, CKD, and diabetes frequently occur together and are linked to worse outcomes, including repeat hospitalisation and accelerated kidney decline. Yet many health systems still organise services around single diseases, creating fragmented pathways and gaps in care. The challenge was to determine whether integrated, nurse-led care could improve clinical outcomes and quality of life for people with advanced multimorbidity.

Approach

The Care HND trial was a randomised study comparing integrated care at the HND Centre with usual care across separate cardiology, nephrology, and diabetes clinics. Adults with established CVD, CKD, and type 1 or 2 diabetes were recruited. A cross-over to integrated care was allowed after one year if patients preferred.

The primary outcome was the first major adverse renal or cardiovascular event (MARCE) over two years (heart failure readmission, myocardial infarction, coronary intervention, stroke, end-stage kidney disease, or death). Person-centred outcomes – including perceived quality of care and empowerment – were assessed using the RAND-36 and EQ-5D-3L at baseline, 6 months, and 12 months. The model relied heavily on nurse-led coordination, patient education, and close communication across specialties.

Results

The trial **(N = 260)** showed a trend towards fewer major renal or cardiovascular events in the integrated care group, particularly fewer heart failure hospitalisations. **In subsequent studies (N = 131)**, patients with heart failure benefited most (hazard ratio 0.55; P = .016). Quality-of-life scores improved in role-physical and social functioning (RAND-36), and EQ-5D visual analogue scale scores increased. Patients also reported better communication and easier access to staff. Overall hospitalisation rates were similar in both groups, but integrated care was feasible and cost-efficient.

Lessons and recommendations

Integrated, nurse-led care is implementable and acceptable for people with advanced MLTC. While effects on hard outcomes may be limited in highly complex populations, coordinated teams can improve patient experience, quality of life, and communication across specialties.

Key lessons:

- Nurse-led coordination strengthens continuity and reduces fragmentation.
- Person-centred training across all staff supports consistent, holistic care.
- Quality-of-life gains may be more sensitive to integrated care than are hospitalisation metrics.
- This model is feasible to scale in settings aiming to improve care for patients with overlapping CVD, CKD, and diabetes.

***Reference***: *Gudrun Evén, Terese Stenfors, Stefan H Jacobson, Tomas Jernberg, Åsa Franzén-Dahlin, Susanna Jäghult, Thomas Kahan, Jonas Spaak, Integrated, person-centred care for patients with complex cardiovascular disease, diabetes mellitus and chronic kidney disease: a randomized trial*, Clinical Kidney Journal, *Volume 17, Issue 11, November 2024, sfae331*, https://doi.org/10.1093/ckj/sfae331

#### ii. System efficiency and safety

Integrated care is not only beneficial for patients, it also enhances the efficiency, safety, and sustainability of health systems (72). By improving coordination among healthcare professionals and avoiding the duplication of diagnostic tests, consultations, and treatments, integrated care reduces waste and makes better use of limited healthcare resources. It also supports continuity of care – particularly crucial for people with chronic or complex conditions – by ensuring that transitions between services and settings are seamless and based on shared information.

One of the clearest system-level advantages is the reduction in unnecessary hospital admissions, emergency department visits, and avoidable complications. People managed through integrated care models typically experience more effective discharge planning, closer post-discharge follow-up, and improved education about their conditions, all of which contribute to reduced readmission rates (73). These benefits are not only clinically important but also have significant financial implications, lowering costs for both healthcare systems and people as well as reducing the travel and logistical burdens placed on families and caregivers (74).

The process of deprescribing, supported by integrated care teams, is another major contributor to system safety and patient wellbeing. By regularly reviewing medication regimens across disciplines, clinicians can identify and discontinue unnecessary or potentially harmful drugs, particularly in elderly persons or those with polypharmacy (75). This leads to simplified treatment plans, better adherence, and fewer adverse drug events, ultimately contributing to safer care.

Integrated care also strengthens the system’s resilience. During health crises such as the COVID-19 pandemic, integrated models enabled remote monitoring, teleconsultations, and continued support for people with chronic conditions despite restrictions on in-person care. Studies have highlighted how these systems were better able to maintain continuity of care and protect vulnerable patients during periods of disruption (75,76).

Beyond immediate efficiency gains, integrated care supports more strategic use of resources through coordinated planning, enabling services to meet population health needs more effectively. The long-term sustainability of health systems – particularly in the context of ageing populations and increasing MLTC – depends on moving away from siloed disease-based care towards integrated, preventive, population-health focused, as well as person-centred models. Table 2 consolidates the abovementioned ways that integrated care improves patient and system outcomes.

**Table 2:** Impact of integrated care on patient and system outcomes.


DOMAIN	OUTCOME	IMPACT OF INTEGRATED CARE	EXAMPLES

Patient experience & quality of care	Care coordination	Improved	Single care plan, shared records, reduced duplication and contradictions.

	Patient satisfaction	Higher satisfaction	Better communication, clearer responsibilities, smoother transitions between services.

	Person-centred care	Enhanced	Care aligned with patient goals, values, and capacity; stronger involvement in decisions.

Clinical outcomes	Disease control	Improved	Better BP control, glycaemic control, lipid management through coordinated follow-up.

	Complication rates	Reduced	Earlier detection and prevention across conditions (e.g., diabetes–CVD interactions).

	Mortality / hospital mortality	Lower or stabilised	Particularly when multidisciplinary teams manage high-risk patients.

Treatment burden & patient capacity	Burden of treatment	Reduced	Streamlined appointments, unified advice, fewer conflicting lifestyle or medication instructions

	Medication burden	Optimised	improved deprescribing practices and reduced polypharmacy in MLTC populations.

	Ability to cope	Increased	Better continuity, coaching, and support from care coordinators and nurses

Healthcare use	Unplannedhospitalisations	Reduced	Coordinated follow-up and early interventions reduce avoidable admissions.

	Emergency department use	Reduced	Better symptom recognition and access to appropriate alternatives.

	Length of stay	Reduced	Earlier discharge planning and better community care.

System efficiency & costs	Duplication of tests & consultations	Reduced	Shared records and multidisciplinary reviews.

	Overall healthcare costs	Costs may decrease or stabilise	Savings from fewer admissions and complications; upfront investment often required.

	Workforce efficiency	Improved	Role optimisation across care teams; reduced fragmentation and task repetition.

Equity & access	Access toappropriate services	Improved	Navigation support for vulnerable groups; more consistent pathways.

	Health disparities	Potentially reduced	Coordinated models can address gaps affecting underserved populations.


#### iii. Policy and practice implications

While the benefits of integrated care are well-established, realising these gains at scale requires substantial policy and system reform. Health systems must address structural and regulatory barriers that impede collaboration across disciplines and services. In many settings, current incentives, funding mechanisms, and legal frameworks are still aligned with fragmented care delivery, making it difficult to embed integrated approaches in routine practice.

Policy priorities should include aligning incentives for interdisciplinary collaboration, modernising regulations to allow for task-sharing and team-based care and developing performance metrics that reflect patient experience and outcomes across MLTC. Embedding the assessment of treatment burden – through tools such as the TBQ – into standard clinical workflows is essential for ensuring care plans are not only evidence-based but also feasible for individuals to follow.

Digital infrastructure must be strengthened to enable data-sharing and care coordination. Investment in interoperable electronic health records, digital patient portals, and remote monitoring platforms can support integrated care delivery while enhancing transparency and communication (78,79). Policymakers should also promote training programmes that equip healthcare professionals with the skills and competencies needed to work effectively in multidisciplinary teams and facilitate patient self-management.

In HICs, the emphasis may be on refining existing models: bridging specialist silos, improving polypharmacy management, and scaling digital solutions. In contrast, LMICs often require lower-cost, high-impact interventions such as task-shifting to community health workers (80), expanding access to mobile technologies, and integrating chronic disease management into existing primary care structures (81). For these settings, flexibility, simplicity, and adaptability are crucial (82).

Importantly, integrated care must be guided by both a population health approach and person-centredness (45). This means aligning care delivery with individuals’ goals, values, and everyday realities, and recognising the dynamic interplay between treatment burden and patient capacity. By embedding this understanding into clinical pathways, funding models, and quality frameworks, integrated care can become a vehicle for health equity as well as clinical excellence (61).

**Integrated care is more than a technical or organisational innovation; it is a moral imperative to deliver care that is not only effective but humane**.

Without coordinated, capacity-sensitive care, people with CVD and MLTC risk poorer outcomes, higher costs, and diminished quality of life. Integrated care offers a path towards a more responsive, resilient, and just healthcare system, one that supports people in managing complex conditions without being overwhelmed by them.

## III. Designing integrated models for CVD and MLTC

### 1. Existing integrated models

**Figure 3:**
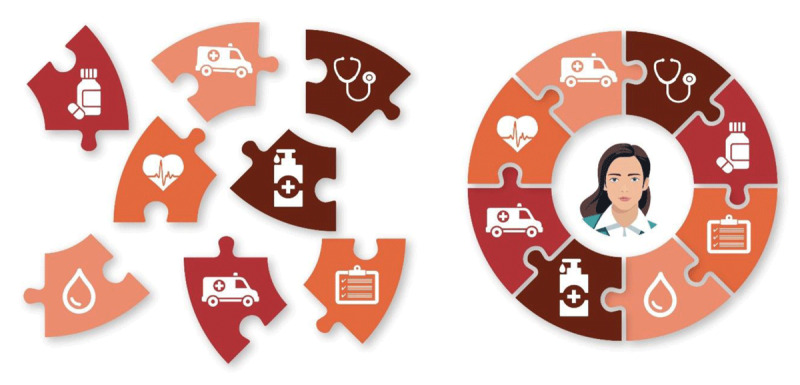
Fragmented vs. Integrated Care Pathways for a Patient. The left panel illustrates siloed, disease-centred services delivered independently across the health system. The right panel demonstrates an integrated, person-centred model in which services are coordinated around the individual, improving continuity, efficiency, and health outcomes.

People living with CVD and MLTC often navigate highly fragmented health systems (Figure 3). These structural shortcomings have prompted the development of integrated care models, which offer a more coherent, person-centred approach to managing complex health needs.

Over the past two decades, numerous models have emerged in response to growing system demands and patient complexity (83). These models vary in scale, structure, and health system context, but consistently emphasise decentralisation, task-shifting, and active patient involvement (84).

Primary care-led approaches, especially in low-resource settings, can employ standardised care protocols and simplified diagnostic algorithms to manage coexisting conditions such as hypertension, diabetes, and CKD (85). For example, the HEARTS in the Americas programme in Trinidad and Tobago illustrates how primary care infrastructure, supported by tailored workforce deployment and service design, can deliver integrated CVD management (86).

As discussed in Section II.1, MASLD is common among people with cardiometabolic disease and is frequently under-recognised in cardiovascular settings, despite CVD being the leading cause of death in this population. To address this gap, an international panel led by Chew et al. developed consensus recommendations to integrate MASLD into cardiovascular care using a modified Delphi approach (87).

The guidance defines practical quality standards for integration including:

- Routine MASLD screening in high-risk cardiovascular populations.
- Incorporation of MASLD into cardiovascular risk assessment.
- Use of non-invasive tests for stratification and monitoring.
- Coordinated management across cardiology, hepatology, endocrinology, dietetics, and nursing.

This example illustrates how integrated care models can embed new diagnostic and management pathways within cardiovascular services, moving beyond single-disease silos to reduce missed opportunities for prevention and improve long-term outcomes.

### 2. The SELFIE framework

**Figure 4:**
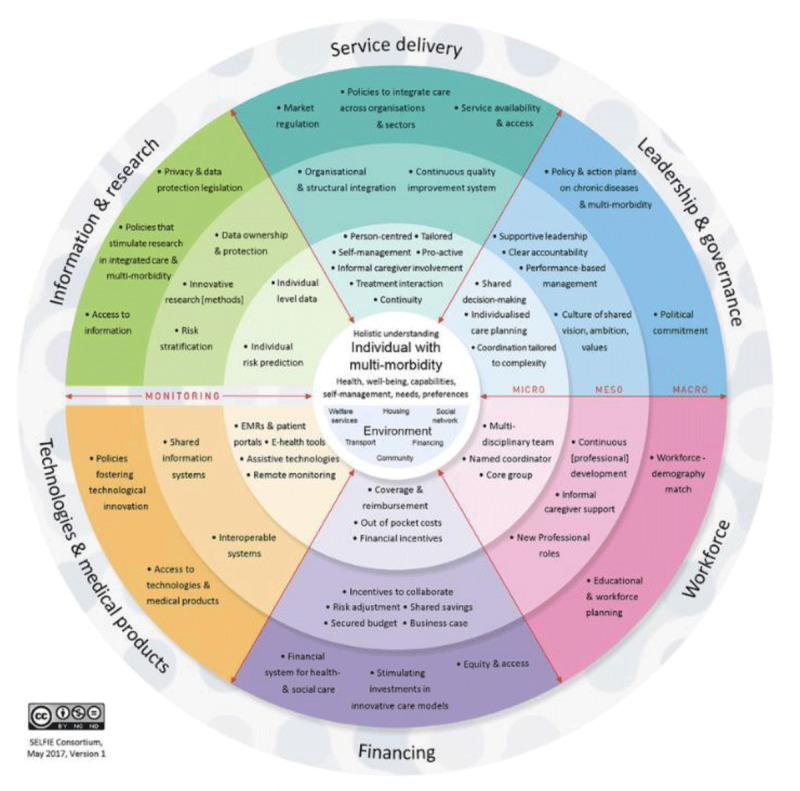
The SELFIE framework for integrated care for multi-morbidity. The SELFIE framework places the individual with multimorbidity at the centre and organises integrated care across micro, meso, and macro levels within six health system domains: service delivery, governance, workforce, financing, technologies, and information systems. It emphasises coordinated, person-centred care supported by aligned policies, resources, and continuous monitoring. The SELFIE framework for integrated care for multimorbidity. Reproduced from Leijten et al. (2018) under the terms of the Creative Commons Attribution 4.0 International License (CC BY 4.0). Reference: https://www.researchgate.net/figure/The-SELFIE-Framework-for-Integrated-Care-for-Multi-Morbidity_fig1_317723529.

To guide the design and evaluation of integrated care for people with multimorbidity, including (Figure 4) CVD and metabolic conditions, the SELFIE (*Sustainable intEgrated care modeLs for multi-morbidity: delivery, FInancing and performancE*) framework offers a robust conceptual structure (88).

It outlines six core components:

1. Service delivery: ensures care is accessible, continuous, and coordinated.
2. Workforce: involves training and deploying multidisciplinary teams.
3. Leadership and governance: aligning institutional roles and policy support.
4. Financing: supporting long-term sustainability through aligned incentives.
5. Technologies and medical products: enabling care through interoperable digital tools.
6. Monitoring and evaluation: measuring outcomes to inform quality improvement.

The SELFIE framework has been successfully applied across high-, middle-, and low-income settings. In Asia, it has been used to map integrated programmes and identify key facilitators such as leadership and digital tools (89). In Europe, the framework has informed economic modelling and implementation guidance for sustainable, person-centred models.

Hospital-based heart failure clinics exemplify this approach by integrating cardiologists, nurses, diabetes educators, dietitians, and mental health professionals to deliver coordinated care and prevent readmissions. These clinics often use digital platforms for remote follow-up, self-management, and shared care planning (43).

However, a seventh component is necessary: person-centredness requires partnership with patients, caregivers, and patient associations. Their co-leadership in designing and evaluating care ensures services are relevant and responsive.

### 3. Key components of integrated care

**Figure 5:**
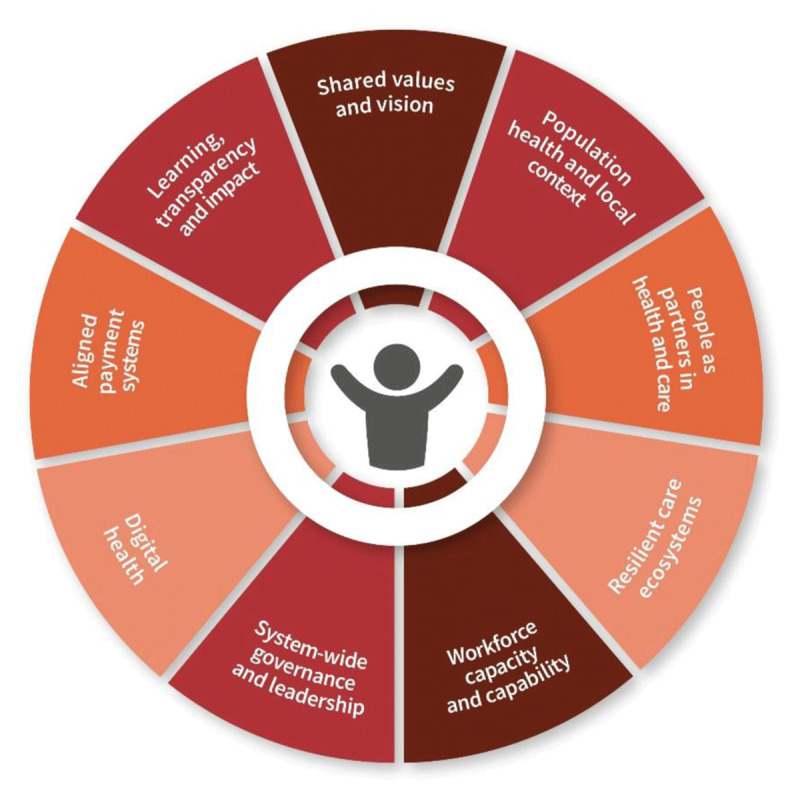
The Key Components of Integrated Care. Integrated care for people living with CVD and MLTCs requires coordinated action across nine interconnected system domains. At the centre is the person, whose needs and capacity should shape care design and delivery.

Designing effective integrated care models (Figure 5) for CVD and MLTC involves a constellation of interrelated components. The *International Foundation for Integrated Care* identifies nine pillars that underpin effective integrated care systems (90):

1. Shared values and vision: A successful integrated care system is built on a clearly articulated and collectively supported vision that prioritises person-centred care, equity, and sustainable improvement. This shared foundation allows diverse stakeholders to align their goals and collaborate effectively.
2. Population health and local context: Care models must be responsive to the specific demographic, cultural, and epidemiological characteristics of the population. Understanding the local disease burden, health behaviours, and service access issues is essential for designing relevant and targeted interventions.
3. People as partners in health and care: Patients, families, and carers are recognised as equal partners in designing, delivering, and evaluating care. This principle supports shared decision-making, self-management, and the development of services that reflect the lived realities of service users.
4. Resilient communities and new alliances: Integrated care extends beyond the health sector, requiring collaboration with education, housing, social services, and civil society to address social determinants of health and build community capacity to support wellness and prevention.
5. Workforce capacity and capability: Health and care professionals need the right mix of skills, training, and support to work collaboratively in multidisciplinary teams. Interprofessional education, new roles such as care coordinators, and team-based service delivery are fundamental enablers.
6. System-wide governance and leadership: Strong leadership is required to guide implementation, overcome institutional silos, and maintain accountability. Governance structures should enable strategic oversight and cross-sectoral alignment.
7. Digital health: Digital solutions, such as interoperable health records, telehealth, and patient apps, facilitate information-sharing and continuity. These are essential enablers of integrated care but also raise equity and governance challenges. A more detailed discussion of digital health, including artificial intelligence (AI), is provided in Section IV.5.
8. Aligned payment systems: Financial mechanisms should incentivise collaboration, prevention, and long-term outcomes rather than episodic or volume-based care. Models such as bundled payments and shared savings are examples of alignment.
9. Transparency of progress, results, and impact: A culture of continuous improvement is driven by open reporting, robust evaluation frameworks, and the use of outcome and process indicators to measure and share results.

At their core, integrated care models are powered by multidisciplinary teams. Working in collaboration, they deliver holistic care that addresses both clinical and psychosocial dimensions of health. In lower-resource contexts, community health workers serve as essential contributors, delivering screening, lifestyle counselling, follow-up, and referral services. For CHWs to be effective, structured training, supervision, and access to mobile or AI-assisted tools are critical (91).

**Case study: Community health worker-led hypertension screening in Kenya**

Context

Non-communicable diseases are the leading cause of premature mortality worldwide, with 75% of these deaths occurring in low- and middle-income countries. In Kenya, hypertension is a major modifiable risk factor for cardiovascular disease. Many people living with hypertension also experience MLTC. National data from 2015 showed a hypertension prevalence of 25% in men and 24% in women, yet 56% of adults had never been screened. Early detection is essential to prevent CVD, but screening coverage remained low. To address this gap, the Healthy Heart Africa (HHA) programme implemented one of Kenya’s largest community-based hypertension initiatives from 2015–2018, aiming to increase screening, awareness, and linkage to care.

Problem

Nearly 90% of adults with hypertension were not receiving treatment, and more than half of Kenyans aged 18–69 had never had their blood pressure measured. The challenge was to develop a scalable approach that could expand screening reach, identify individuals with elevated blood pressure early, and link them effectively to diagnosis and treatment.

Approach

The programme trained CHWs using the Kenya Ministry of Health curriculum and deployed them to conduct both mass and opportunistic hypertension screening. At health facilities, all patients attending for any service were screened. In communities, CHWs carried out screening in households, busy public spaces, and religious institutions. Individuals with raised blood pressure were referred to a nearby facility for confirmation, diagnosis, and treatment. CHWs also followed up patients identified in the community to support linkage to care. This model was chosen because CHWs are trusted local actors, able to reach large numbers of people and provide culturally appropriate health education.

Results

Across the programme period, 663,028 blood pressure screenings were completed, with 73.9% conducted in community settings. A total of 66,981 people were started on treatment, most of whom were women (71.2%), with a median age of 55 years. While the majority of screened individuals were young adults (18–34 years), the model successfully identified large numbers of people requiring follow-up and treatment.

Lessons and recommendations

This initiative demonstrated that CHW-led screening is feasible at scale and can significantly expand access to hypertension detection.

Key lessons:

- CHWs can substantially increase reach through both community and facility-based approaches.
- Clear referral and follow-up pathways are essential to ensure linkage to treatment.
- Younger adults are frequently screened, but targeted strategies are needed to reach higher-risk groups who may be underrepresented in community campaigns.

Embedding CHW-led screening within national programmes can support sustainable hypertension control efforts.

***Reference:*** *Mbau L, Harrison R, Kizito W, Timire C, Namusonge T, Muhula S, Nyanjau L, Owiti P. Case identification, retention and blood pressure control in Kenya. Public Health Action. 2022 Jun 21;12(2):58–63. doi: 10.5588/pha.21.0051. PMID: 35734002; PMCID: PMC9176190*.

Digital tools and data systems are central to enabling integration. Electronic health records, health information exchanges, and mobile health applications allow for real-time communication and monitoring. Technologies such as wearable devices, AI-supported analytics, and virtual consultations can help predict risk, enhance access, and personalise care (92,93). Nonetheless, barriers remain. Fragmented IT infrastructure, along with regulatory and privacy concerns, can impede data-sharing. Solutions must prioritise interoperability, equitable access, and user-friendly features.

Finally, organisational and financial structures must support integration through appropriate governance, incentives, and monitoring systems (94,95). Capitation or bundled payment models, when aligned with quality outcomes, can promote coordination and sustainability (96). Governance frameworks should include leadership structures, multidisciplinary representation, and mechanisms for feedback and continuous learning.

## IV. Implementation strategies for integrated care models

Delivering integrated care for people living with CVD and MLTC requires more than designing the right components. It also demands thoughtful, context-sensitive implementation.

Both clinician and patient WHF surveys pointed to potential solutions: patients called for better communication, financial support, and access to multidisciplinary teams, while healthcare professionals highlighted the need for patient education, simplified referral systems, and digital monitoring tools. Importantly, inclusion of integrated care within national health policies was considered essential by both groups. These insights reinforce the need for system-level reform to make integrated cardiovascular care feasible and sustainable worldwide.

**Voices from patients and clinicians on integrated care**

To inform this roadmap, the WHF conducted two global surveys:

1. Healthcare professionals (n = 78): majority were cardiologists, with broad geographic representation.
  Most common comorbidities seen diabetes (56%), obesity (20%), CKD (13%).
  Barriers: insufficient funding (74%), fragmented systems (64%), lack of political prioritisation (64%).
  Solutions: patient awareness, training for healthcare professionals, national policies, accessible and affordable medicines, digital monitoring.
2. Patients with CVD and carers (n = 58): 98% were patients living with CVD, often with multiple conditions (hypertension 50%, diabetes 48%, mental health 28%).
  76% took five or more medications.
  57% reported poor coordination of care and 55% long waits or distance to services.
  Only 9% had access to integrated risk assessment tools.
  Priorities: better communication, financial support, access to multidisciplinary teams.

Key takeaway: Both groups strongly support integrated care, but systemic, financial, and organisational barriers continue to limit implementation. Their voices highlight the urgency of building sustainable models of care that bridge these gaps.

Implementation must align with local priorities, capacities, and health system structures, while staying true to the shared goal of person-centred, equitable, and coordinated care (97).

### 1. Policy and governance

Strong governance and policy frameworks create the foundation for integrated care. As discussed earlier (see Section III.3: System-wide governance and leadership), effective integration relies on shared values, strategic alignment, and collaboration across sectors. Enabling legislation can formalise multidisciplinary, team-based approaches and clarify roles and responsibilities at national, regional, and local levels.

Leadership should be multisectoral and inclusive, engaging key actors, such as health authorities, professional organisations, patient advocacy groups, and civil society. Tools such as the policy triangle – considering context, content, and actors – can help identify policy barriers and guide the development of cohesive strategies.

### 2. Financing and resource allocation

Financial mechanisms must support, not undermine the goals of integrated care. As outlined under aligned payment systems (Section III), traditional fee-for-service models are poorly suited to the complexity of MLTC. Alternative approaches such as bundled payments, capitation, or shared savings arrangements can incentivise coordination and continuity of care (98).

Equally important is investment in the infrastructure needed to support integrated services, particularly digital health tools and community-based delivery platforms. In LMICs, where public funding may be constrained, donor assistance and external technical support can play a catalytic role (99), but sustainability depends on resilient, locally owned financial frameworks.

### 3. Workforce development

A capable and supported workforce is the engine of integrated care. As noted in Section III.3, multidisciplinary teams must be equipped with the skills to collaborate across disciplines and manage people with complex needs. This requires both initial training and ongoing professional development in areas such as chronic disease management, team-based care, shared decision-making, and the use of digital tools. Examples include interprofessional rounds involving medicine, nursing, pharmacy, and social workers, mandatory primary care rotations for specialists, and continuing medical education modules on MLTC recognition or comorbidity screening.

At a system level, education and accreditation levers such as embedding MLTC competencies within medical curricula, assessments, and continuing education requirements, could help ensure that integrated care principles are consistently reinforced across the workforce.

Task-sharing is particularly relevant in resource-constrained settings. CHWs, nurse practitioners, patient navigators and care coordinators can extend the reach of integrated care when properly trained and supported. (See also Section III: CHWs and non-physician roles.)

### 4. Patient engagement and education

Integrated care must be co-designed with individuals and communities. Earlier we emphasised the role of patients as partners in care (Section III), highlighting shared decision-making and culturally appropriate engagement strategies. Implementation efforts should include investment in health literacy (particularly important as people increasingly encounter misleading health information online) development of multimedia self-management tools, and support for family and peer involvement. Patients should also be empowered to become advocates for better healthcare within their communities.

Importantly, systems must also create feedback loops – mechanisms for people to express needs, preferences, and experiences – which can inform service improvement and ensure care remains responsive.

### 5. Digital infrastructure and data systems

Digital health is a cornerstone of implementing integrated care for people with CVD and MLTC. Building on the *WHF Roadmap on Digital Health in Cardiology* (77), which outlined opportunities and challenges for digital transformation, this roadmap expands the perspective to multimorbidity and highlights the critical role of technology in implementation.

Beyond basic IT infrastructure, digital tools can transform care delivery, support patient engagement, and enable more equitable and person-centred models (100,101).

Integrated EHRs, health information exchanges, and patient portals allow for continuity across healthcare professionals, providers, settings (and eventually borders, in Europe), reducing duplication and facilitating shared decision-making (45,86). Mobile health and telemedicine extend the reach of services into communities, particularly in rural and underserved areas (102). Wearables and remote monitoring tools enable real-time tracking of symptoms, treatment adherence, and disease progression, allowing timely interventions and reducing avoidable hospitalisations (103,104).

Artificial intelligence and advanced analytics add a further dimension. Predictive models can identify individuals at risk of deterioration, hospitalisation, or treatment non-adherence (104,105). Natural language processing can extract relevant information from clinical records (106,107), and AI-driven decision support can assist health workers with complex multimorbidity management (108). In resource-constrained settings, simplified mobile tools and AI-assisted training for community health workers can help bridge workforce gaps (109,110).

However, digital transformation also brings challenges. Interoperability between systems remains limited, even in HICs (109,111). Data privacy, ownership, and algorithmic bias raise ethical concerns (77,112), while the “black box” nature of some AI models undermines transparency and trust. The digital divide (driven by age, literacy, socioeconomic status, and geography) risks excluding those most in need of integrated care.

To ensure safe, equitable, and sustainable adoption, countries must:

- Invest in interoperable digital infrastructure aligned with national health strategies.
- Build robust governance frameworks for data security, consent, and algorithm oversight.
- Promote inclusive design and co-creation with patients and caregivers, ensuring tools are usable and context appropriate.
- Strengthen digital literacy and workforce training, enabling clinicians, CHWs, and patients to make effective use of technology.
- Embed digital health evaluation in learning health systems (see Section VII) to monitor effectiveness, safety, and equity over time.

If implemented responsibly, digital health and AI can be powerful enablers of integrated care, improving continuity, personalisation, and efficiency while reducing treatment burden and health inequities.

### 6. Monitoring, evaluation, and learning health systems

Finally, the implementation of integrated care must be guided by robust monitoring and evaluation. This connects directly to the pillar of transparency and continuous improvement (Section III). M&E systems should be embedded from the beginning, with clear indicators that measure outcomes, quality, patient experience, and equity.

Adopting a learning health system approach (113) – where real-time data feeds continuous improvement – can make implementation more adaptive and evidence-driven. Transparent reporting of progress to stakeholders fosters accountability, strengthens community trust, and helps ensure that integrated care models are delivering meaningful benefits over time.

**Figure 6:**
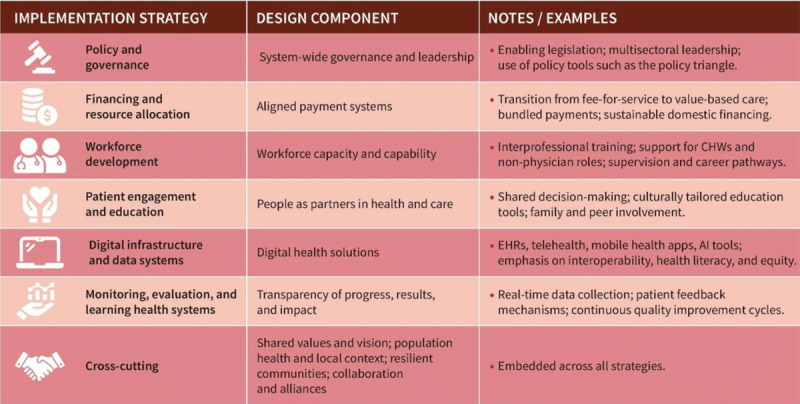
Implementation Strategies and Design Components. Core implementation strategies, associated structural and functional design components, and practical examples to support effective, equitable, and sustainable delivery of integrated care.

By adopting these strategic components – rooted in strong policy, sustainable financing, empowered healthcare professionals, and engaged (Figure 6) patients – health systems can build integrated models that are not only clinically effective, but equitable, resilient, and responsive to the complex realities of people living with CVD and MLTC.

## V. From barriers to breakthroughs: roadblocks and accelerators to integrated care

Designing integrated care models and defining implementation strategies is not enough.

Many initiatives struggle to achieve scale or sustainability despite strong evidence, policy endorsement, and professional support. This section examines factors that determine whether integrated care moves from concept to impact, by focusing both on roadblocks and system-level accelerators that shape implementation.

Across settings, successful integrated care initiatives share a common set of facilitating conditions that cut across governance, financing, workforce, digital infrastructure, and patient engagement.

### 1. Why integrated care stalls in practice: key roadblocks

Fragmentation remains the most frequently reported obstacle by both healthcare professionals and patients.

#### i. Fragmented governance and financing

Separate funding streams and misaligned incentives that prioritise episodic, volume-based care, as well as siloed services and poorly coordinated referral pathways lead to duplication, inconsistent advice, and avoidable hospitalisations.

WHF survey respondents highlighted insufficient funding, lack of political prioritisation, and fragmented delivery as dominant barriers, laying bare the gap between political ambition and operational reality.

#### ii. Infrastructure and digital fragmentation

In many LMICs, limited physical infrastructure, unreliable supply chains and workforce shortages constrain implementation. In HICs, digital fragmentation and poor interoperability undermine continuity, despite technological advances.

The digital divide, which runs along socioeconomic and geographical lines but is also shaped by age and literacy, risks excluding those most affected by MLTC.

#### iii. Workforce shortages and skills misalignment

Medical and nursing education continues to emphasise single-disease models, leaving many professionals unprepared to manage MLTC and their related issues of polypharmacy and treatment burden. Training gaps in communication, shared decision-making, and coordination also weaken integrated care.

High workload, burnout, and workforce attrition compound these challenges across settings.

#### iv. Cultural and organisational resistance

Professional silos, hierarchical structures, unclear accountability, and resistance to role change remain significant roadblocks. Integrated care requires redistribution of authority and responsibility, which may generate uncertainty or opposition among clinicians and institutions.

People with low health literacy or mistrust of health systems may also struggle to navigate multidisciplinary models without support.

#### v. Inadequate measurement, evaluation, and learning systems

Many health systems lack appropriate metrics to capture the complexity of integrated care for MLTC. Disease-specific indicators dominate evaluation, while PROMs, treatment burden, and equity metrics are undervalued.

Without embedding monitoring and feedback loops, programmes struggle to adapt, learn, or demonstrate value, limiting scale-up and sustainability (see section VI).

**Integrated care requires systems that are configured to support collaboration, continuity, and focus on both population health and person-centred outcomes**.

**Addressing accelerators and roadblocks simultaneously – across policy, financing, workforce, digital infrastructure, and evaluation – is essential to translate integrated care from pilot projects into practice**.

### 2. Key accelerators to facilitate integrated care

Key accelerators translate the principles of integrated care into practical system change by shaping how services are funded, organised, and rewarded (114).

#### i. Alignment of incentives with long-term, person-centred outcomes

The shift towards integration is most likely to succeed when financial and organisational incentives reward continuity, collaboration, and prevention, rather than episodic, volume-based activity (115). Payment models such as capitation, bundled payments, or shared savings create space for multidisciplinary teamwork, proactive follow-up, and coordinated care, all of which are essential for managing MLTC.

Where incentives remain fragmented, integrated services (non-visit-based activities) such as care coordination, patient education, or medication review are often underfunded or excluded altogether, limiting sustainability. Alignment of financing with integrated care objectives is a fundamental accelerator (see sections III and IV.2).

#### ii. Legal and regulatory frameworks that support team-based care

Supportive regulatory frameworks allow expanded scopes of practice to enable task-sharing and accountability across multidisciplinary teams. Countries that permit nurse-led clinics, pharmacist-managed medication reviews, and CHW involvement are better positioned to deliver integrated care at scale.

Clear definitions of roles and responsibilities, liability arrangements, and professional recognition of new and evolving functions are particularly important in resource-constrained settings where task-sharing is essential for access and continuity (see sections III and IV.3).

#### iii. Digital infrastructures embedded within governance and care pathways

Digital health is not just about technology, it is a system issue. Digital infrastructures that are government-enabled and workflow-embedded are powerful accelerators of care integration. Technology can facilitate information continuity, shared decision-making, and real-time learning. Interoperable electronic health records, patient portals, telemedicine, and remote monitoring tools support coordination between healthcare professionals and across care settings.

However, technology alone does not create integration. Successful programmes embed digital tools within governance frameworks, workforce training, and evaluation systems, ensuring usability, equity, and data protection (see section IV.5).

#### iv. Workforce capability, legitimacy, and collaborative culture

Integrated care depends on a workforce that is trained, supported, and culturally prepared to work outside of traditional boundaries. Interprofessional education, team-based workflows, trust across disciplines, and shared accountability foster collaboration and reduce silos.

Equally important is legitimacy. Healthcare professionals must trust new roles, value collaboration, and perceive integrated care as improving – rather than threatening – service quality. Where professional hierarchies remain rigid, implementation is often resisted (see sections III and IV.3).

#### v. Active patient, caregiver, and community engagement

Integrated care is more effective when people living with CVD and MLTC are engaged alongside their caregivers and communities as partners rather than passive recipients of health services. Health literacy initiatives, shared decision-making, peer support, and caregiver involvement strengthen self-management and adherence (116,117).

Patient engagement also improves system responsiveness by ensuring that care models reflect lived experience, treatment burden, and individual capacities (see sections II and IV. 4) (118).

#### vi. Embedded monitoring, evaluation, and learning systems

Integrated care is more likely to be sustained and scaled when monitoring and evaluation are embedded within routine practice and linked to continuous learning. The use of meaningful, person-centred and equity-sensitive indicators (beyond disease-specific metrics) allows health systems to assess whether integrated care is improving lived experience, treatment burden, continuity, and outcomes for people with CVD and MLTC (119).

Continuous feedback loops support adaptation and improvement over time, while evidence generated through learning systems strengthens accountability, informs investment decisions, and reinforces political commitment. Embedding these functions within integrated care governance creates the foundation for effective implementation and long-term impact (see Section VI).

**Figure 7:**
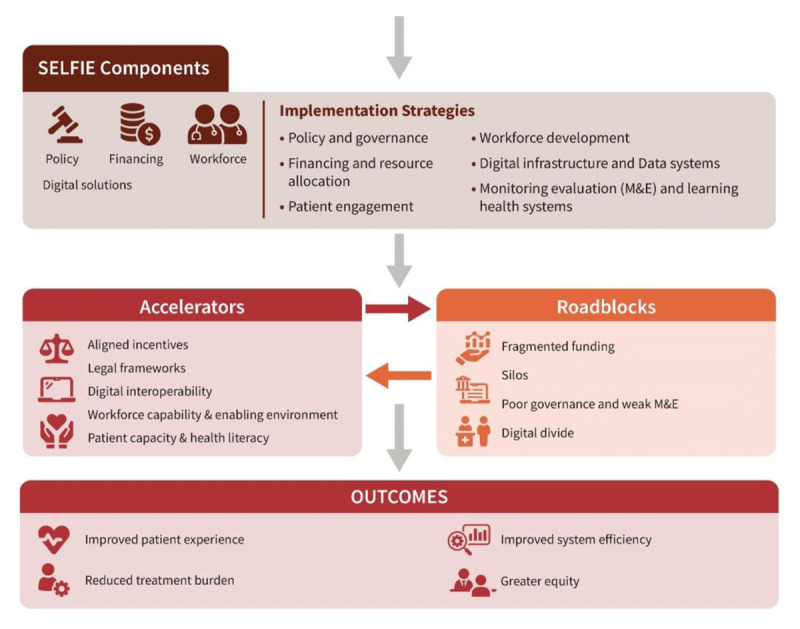
From Design to Impact: Why Integrated Care Succeeds or Fails. The framework demonstrates how foundational system components translate into implementation strategies, which are either facilitated or hindered by contextual accelerators and barriers, ultimately determining patient-level and health system-level outcomes.

Figure 7 is a framework that demonstrates how implementation strategies can address barriers to improve patient-level and health system-level outcomes.

## VI. Implementation in diverse settings

### 1. High-income vs. low- and middle-income contexts

While the principles of integrated care are applicable globally, their implementation must be tailored to the realities of local health systems. As mentioned previously, HICs and LMICs face distinct structural and resource-related challenges, requiring different strategies for success (120).

In HICs, challenges often relate to system complexity and fragmentation. Care is frequently delivered through highly specialised services with limited coordination, resulting in duplicative diagnostics and inconsistent patient follow-up. Data fragmentation and siloed communication systems further hinder continuity. Common strategies to mitigate these issues include the use of shared digital health platforms, multidisciplinary case conferences, and pharmacist-led medication reviews. PROMs are increasingly used to personalise and track care over time (121).

In contrast, LMICs typically face constrained health workforces, under-resourced primary care systems, and limited access to diagnostics and specialist care (122). Weak referral networks and high out-of-pocket costs exacerbate barriers to care. Yet, innovative models based on community outreach, task-shifting to non-physician healthcare providers, and the use of mHealth tools offer promising alternatives. Community health workers play a critical role in delivering basic screening, health education, and chronic disease monitoring (123). Simplified care algorithms are used to support frontline decision-making (124).

Despite differing contexts, both HICs and LMICs benefit from strong leadership, engaged communities, adaptable models, and robust systems for performance monitoring and learning. National strategies that support decentralised planning and flexible service delivery while retaining clear quality benchmarks can be adapted to suit resource levels.

### 2. Community-based strategies

Community-based actions are essential to reach individuals with CVD and MLTC who might otherwise be excluded from traditional health systems. These strategies are particularly critical in LMICs and underserved regions of HICs, where access to health facilities and specialists may be limited.

CHWs, when adequately trained and supported, are instrumental in expanding the reach of integrated care (125). They can carry out screening for hypertension, diabetes, and mental health conditions; provide patient and caregiver education; and monitor medication adherence and symptom progression. To be effective, these roles must be backed by clear protocols, supervisory frameworks, and appropriate remuneration (126).

Community-based health promotion and prevention initiatives integrated into settings such as schools, faith groups, and local associations further amplify the reach and sustainability of care. Participatory approaches that include patients and caregivers in programme design foster engagement and enhance adherence.

Training is a foundational facilitator. Interprofessional education is pivotal to team-based care, while targeted instruction for CHWs and nurses ensures safe and effective task-shifting. Decision-support tools, culturally sensitive materials, and ongoing mentorship help maintain quality across diverse populations.

### 3. Role of digital health

Digital health tools, including mHealth, telemedicine, EHRs, and AI-driven analytics, are vital enablers across both high- and low-resource settings. Their role in bridging workforce gaps, improving coordination, and supporting self-management is transformative. Key considerations for implementation (equity, interoperability, governance, AI ethics) are discussed in detail in Section IV.5.

Implementing integrated care for CVD and MLTC requires flexibility, contextual awareness, and sustained investment (127). While HICs must overcome fragmentation and siloed care delivery, LMICs need to build systems from limited resources using community and digital innovations.

Across all settings, community-based solutions and digital tools act as vital connectors between patients and coordinated care systems. By embracing context-sensitive implementation strategies grounded in the SELFIE framework and supported by robust policy, training, and engagement, integrated care models can be scaled to meet the complex needs of diverse populations.

## VII. Monitoring, evaluation, and learning

Monitoring and evaluation are not simply operational add-ons; they are central to building learning health systems that can sustain and continuously improve integrated care for people living with CVD and MLTC (128). Unlike disease-specific programmes, integrated care for MLTC requires balancing complex needs, coordinating across sectors, and adapting to heterogeneity in patient trajectories. Without robust M&E, health systems risk replicating single-disease silos, overlooking unintended consequences, or failing to capture the true value of integrated approaches (129).

M&E in this context serves three purposes:

1. Accountability: demonstrating whether integrated models deliver on their promise of improved outcomes, efficiency, and equity.
2. Learning: generating timely feedback to adapt care models to real-world challenges and diverse populations.
3. Scaling: identifying transferable lessons that can inform system-wide reforms beyond pilots or isolated projects.

### 1. Evaluation frameworks

Structured frameworks are necessary to evaluate the multiple dimensions of integrated care.

Proctor’s implementation evaluation model (130,131) provides a useful structure by distinguishing between:

- Implementation outcomes: adoption, fidelity, acceptability, feasibility, sustainability.
- Service outcomes: effectiveness, efficiency, safety, equity, person-centredness.
- Patient outcomes: health status, quality of life, treatment burden, satisfaction, survival.

**Figure 8:**
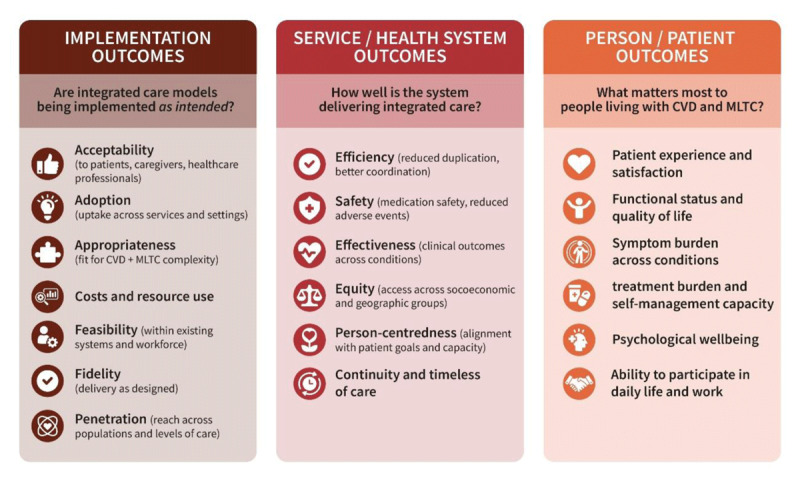
Outcomes Framework for Integrated Care in CVD and MLTC. Framework for evaluating integrated care across implementation, health system, and patient-level outcomes. Adapted from Proctor et al 2011.

This tripartite framing is especially relevant in MLTC because it captures not only whether services are clinically effective, but also whether they are coordinated, acceptable to both patients and professionals, and sustainable across care settings (Figure 8).

### 2. Data and measurement for integrated care

Evaluating integrated care requires linking data across conditions, healthcare professionals, patients, and settings (132). For people with MLTC, outcome measurement cannot be confined to disease-specific indicators (e.g. blood pressure or HbA1c control). It must also include:

- PROs and experience measures (PREMs), reflecting quality of life, treatment burden, and ability to self-manage across conditions.
- Cross-sectoral indicators, such as continuity between primary care, specialist services, and community support.
- Equity metrics, to ensure models benefit disadvantaged populations who are often most affected by MLTC.

High-resource systems may use electronic health records and integrated dashboards, while low-resource contexts may rely on pragmatic tools (mobile checklists, community registers) (133). In both, patient and community participation in selecting outcomes enhances relevance and trust.

### 3. From monitoring to learning health systems

Traditional M&E often produces static reports, but integrated care requires dynamic learning systems that provide timely insights to clinicians, managers, and policymakers (134). A learning health system continuously collects, analyses, and feeds back data to drive improvement (109).

Key elements include:

- Real-time feedback loops to frontline teams.
- Data visualisation tools for shared decision-making.
- Participatory co-design with patients and communities.
- Alignment of evaluation with planning and quality improvement cycles.

These systems are particularly important for CVD and MLTC, where treatment complexity, polypharmacy, and multimorbidity can generate unanticipated outcomes. For example, PROMs can identify where integrated care reduces treatment burden but increases complexity elsewhere, insights invisible to traditional metrics.

**Case study: Using PROMs to redesign care pathways in an urban CVD + MLTC clinic**

Context

This case comes from heart failure clinics at the University Health Network (UHN) in Toronto, Canada. The clinics introduced electronic PROMs, specifically the Kansas City Cardiomyopathy Questionnaire (KCCQ-12), to better understand patients’ symptoms, limitations, and quality of life. PROMs were integrated into routine care using implementation science frameworks: RE-AIM (Reach, Effectiveness, Adoption, Implementation, Maintenance) to assess clinical integration, and the Theoretical Domains Framework (TDF) to explore behavioural factors affecting clinicians’ uptake. This evaluation reflects real-world challenges in embedding PROMs into busy, multidisciplinary services treating people with cardiovascular disease and MLTC.

Problem

Although PROMs offer valuable insight into patients’ lived experiences, their use in routine heart failure care is often inconsistent. Furthermore, HF also is associated with a higher prevalence of depressive symptoms, anxiety, and coexisting cognitive issues. The challenge was to understand how well PROMs were being adopted in the two clinics, why clinician usage varied, and what barriers and facilitators shaped sustained implementation. PROMs needed to fit into workflows without creating additional burden for staff or patients.

Approach

Heart failure patients at Toronto General and Toronto Western Hospitals were assigned KCCQ-12 questionnaires either automatically (for new patients) or manually by clinicians using prompts in the electronic record. Patients completed PROMs through MyChart, a secure digital platform. PROMs were aligned with clinic visits and limited to once every two weeks.

Clinicians – including cardiologists, internists, nurses, and nurse practitioners – were grouped by how often they assigned PROMs. Interviews with representatives from each group explored knowledge, beliefs, workflow barriers, and resource needs, guided by TDF. Clinic data were analysed to assess assignment and completion rates, patient characteristics, and trends in KCCQ-12 scores.

Results

PROM assignment and completion improved over time, reaching 80.7% completion by September 2023. Uptake was higher at Toronto General, and most completed PROMs came from male patients. Around 80% of patients consistently reported high quality of life, although a temporary decline occurred mid-year. Clinician adoption varied widely: only 11% regularly assigned PROMs, while 68% never did. Interviews identified barriers related to confidence, intention, workflow pressures, and emotional factors. Suggested strategies included targeted education, practical training, clearer workflows, and environmental prompts.

Lessons and recommendations

Sustained PROM use requires more than technical implementation.

Key lessons:

- Clear workflows and prompts support routine PROM assignment.
- Clinicians need training and reinforcement to understand how PROMs inform care.
- Behaviour-change strategies – education, modelling, environmental restructuring – can improve uptake.
- PROMs can meaningfully guide care for patients with CVD and MLTC, but adoption depends on embedding tools into everyday practice.

This evaluation shows that PROMs can be integrated into heart failure pathways and provide valuable patient insight but require ongoing support to become part of routine care.

*Reference: Sarah V.C. Lawrason, Heather Ross, Michael McDonald, Juan Duero Posada, Samantha Engbers, Anne Simard, Using Implementation Science to Evaluate the Implementation of Patient-Reported Outcome Measures (PROMs) in a Clinical Heart Failure Care Setting, CJC Open, Volume 6, Issue 12, 2024. Pages 1443–1452, ISSN 2589-790X:* https://doi.org/10.1016/j.cjco.2024.09.012.

### 4. Building capacity for sustainability

Donor-funded or pilot projects should invest in local M&E capacity so that learning systems persist beyond initial funding. This includes training health professionals in data use, standardising indicators, and fostering cultures of learning rather than compliance.

Monitoring and evaluation in integrated care for CVD and MLTC is not about proving a model works in the abstract. It is about learning what works, for whom, and under what circumstances, and using that knowledge to refine practice and policy. By embedding Proctor’s framework within a learning health system approach, health systems can ensure integrated care is not only implemented but continuously improved, equitable, and sustainable.

## VIII. Conclusion and future directions

### 1. Summary of key points

The increasing prevalence of CVD related to MLTC presents one of the most significant challenges to populations and health systems worldwide. Individuals living with these overlapping conditions often face fragmented, burdensome, and inefficient care that fails to meet their complex needs. This roadmap calls for a transformative shift towards integrated care, one that is both person-centred and population health-focused, as well as equitable and responsive to the realities of both patients and health systems.

Integrated care is not simply an ideal: it is a necessity. As highlighted throughout this roadmap, CVD rarely exists in isolation. MLTC are the norm, not the exception, and addressing them through siloed, single-disease pathways is no longer tenable. Integrated care models have been shown to improve quality of life, reduce complications, enhance cost-efficiency, and strengthen the overall resilience of health systems.

This roadmap outlines actionable solutions that can be adapted across diverse settings. It presents a practical vision based on the SELFIE framework, with a focus on multidisciplinary teams, coordinated service delivery, informed use of technology, and continuous monitoring and evaluation. It also emphasises the importance of aligning governmental, legal, financial, and regulatory systems; addressing workforce and training gaps; and recognising the human cost of inaction, particularly among vulnerable populations in low-resource settings.

Equally critical is the concept of patient capacity. Integrated care must consider what people can realistically manage, not just what is medically prescribed. Without this balance, treatment burden increases, adherence declines, and outcomes worsen.

Through a combination of evidence, case examples, and implementation tools, this roadmap provides a comprehensive guide to reconfiguring care for people with CVD and MLTC. It supports healthcare professionals, policymakers, and communities in designing context-sensitive, patient-responsive systems that work.

### 2. A call to action

The time to act is now. Implementing integrated care to face the syndemic of CVD and MLTC requires urgency, vision, and collaboration.

Policymakers must align financing and regulatory frameworks to support integrated care models and enable task-sharing across professions. Investment in primary care, digital health infrastructure, and community-based delivery models is essential, particularly in low- and middle-income countries, where health system fragility amplifies risk.

Healthcare leaders should foster a culture of collaboration and shared accountability. This includes teamwork across disciplines, joint protocols, and robust training in both clinical and person-centred competencies. Clinicians, in turn, need to embrace interprofessional cooperation and actively include patients and caregivers in care planning.

Communities and persons with CVD and MLTC must be recognised as vital stakeholders. Their involvement in designing, monitoring, and refining care is not optional, it is foundational to making integrated care work. Patient voices offer unique insight into the lived experience of navigating chronic illness and provide a compass for improving equity, continuity, and trust.

The COVID-19 pandemic laid bare the weaknesses of health systems globally, particularly in the care of vulnerable populations with chronic conditions. As the world braces for future threats, whether from emerging infections, climate-related shocks, or demographic shifts, integrated care emerges as a critical strategy for strengthening system resilience.

Integrated care is a long-term investment in the dignity, autonomy, and wellbeing of people living with complex health needs.

### 3. Future directions

This roadmap is a living document. It offers not only a strategic framework for action, but also a platform for innovation, learning, and local adaptation.

Several opportunities lie ahead. Research into the effectiveness and cost-efficiency of integrated care models in varied settings is needed. Innovation in digital health, especially open-source platforms and tools for low-resource settings, can accelerate scale-up and support task-shifting. There is also a need for integrated, evidence-based guidelines for the management of CVD and MLTC, to support clinical decision-making, reduce unwarranted variation, and align care across settings and disciplines. Developing standardised care pathways and expanding health information exchange will help ensure continuity and equity.

At the same time, building learning health systems must become a core objective. These systems use real-time data and community feedback to support iterative improvement, policy refinement, and evidence-informed decision-making. As highlighted in the monitoring and evaluation chapter, strong learning systems are crucial to sustaining integrated care.

Ultimately, this roadmap envisions a future in which every person with CVD and MLTC, regardless of geography, income, social status, ethnicity and sexual orientation, receives care that is evidence-based, coordinated, continuous, and compassionate. Achieving that future requires bold decisions, smart investment, and collective commitment. But the reward can be profound: resilient healthcare systems capable of meeting today’s challenges and tomorrow’s uncertainties and, most importantly, better lives for patients, who will experience improved outcomes and more efficient and person-centred care.

**Call to action**

**Integrated care for people living with – or at risk of – CVD and MLTC must be implemented urgently through aligned policies, shared leadership, patient and community engagement, and targeted investment, especially in primary care, to build more equitable, resilient, and person-centred health systems**.

## Annex

### Quick guide: Monitoring and evaluation for integrated care in CVD and MLTC

#### Purpose

To support implementers in establishing practical and context-sensitive systems to monitor and evaluate integrated care programmes for people with CVD and MLTC.

#### Step-by-step guide to getting started

1. **Clarify your goals**
  What are you trying to improve? (e.g. hospital readmissions, quality of life, care coordination).
2. **Choose a framework**
  Use the Proctor model to ensure you evaluate:
  - Implementation outcomes: adoption, fidelity, feasibility, appropriateness, acceptability.
  - Service outcomes: efficiency, timeliness, safety, equity.
  - Patient outcomes: quality of life, symptom control, mortality.
3. **Select indicators (KPIs)**
  Indicators should reflect both performance and person-centred outcomes. Refer to Chapter VI’s indicator table for guidance.
  - Start with a minimal core set, then expand as capacity grows.
  - See core indicators table (Chapter 6) for examples.
  - Plan your data sources:
    - EHRs? Surveys? CHWs?
  - Consider both digital and paper tools.
4. **Involve your team**
  - Engage healthcare professionals, patients, and community reps in selecting and interpreting indicators.
  - Embed M&E into care processes.
  - Use shared dashboards or regular team huddles to review progress. Close the loop.
  - Feed findings back into clinical decisions, policies, and service redesign.
5. **Sample indicator set**
  Tailor your core set to local context and resources. Build progressively.
  **Table:**
  **Category**	**Example KPI**
  Implementation	% of clinics using new integrated care pathway
  Service	30-day readmission rate for people with CVD
  Patient	% of patients reporting reduced treatment burden
6. **Tips for success**
  - Co-design with patients and frontline staff.
  - Use mixed methods: combine numbers with patient and staff interviews.
  - Start small: pilot your approach in one region or team.
  - Plan for equity: disaggregate data by age, gender, income, geography.
  - Make it actionable: don’t collect data you won’t use.
  - Align with planning and budgeting cycles.
  - Visualise results: use simple dashboards to communicate trends.
7. **Building a learning health system**
  *“What gets measured gets improved.”*
  Features of a learning system:
  - Real-time feedback loops.
  - Continuous training and peer exchange.
  - Shared platforms for improvement.
  - Embedded patient voice.

Feedback loop in a learning health system

https://www.allianceon.org/EPIC-Learning-Health-System
